# Oct2 and Obf1 as Facilitators of B:T Cell Collaboration during a Humoral Immune Response

**DOI:** 10.3389/fimmu.2014.00108

**Published:** 2014-03-20

**Authors:** Lynn Corcoran, Dianne Emslie, Tobias Kratina, Wei Shi, Susanne Hirsch, Nadine Taubenheim, Stephane Chevrier

**Affiliations:** ^1^Molecular Immunology Division, Walter and Eliza Hall Institute of Medical Research, Melbourne, VIC, Australia; ^2^Department of Medical Biology, The University of Melbourne, Melbourne, VIC, Australia

**Keywords:** Oct2, Obf1, Syk, Slamf1, B:T collaboration, cytokines

## Abstract

The Oct2 protein, encoded by the *Pou2f2* gene, was originally predicted to act as a DNA binding transcriptional activator of immunoglobulin (Ig) in B lineage cells. This prediction flowed from the earlier observation that an 8-bp sequence, the “octamer motif,” was a highly conserved component of most Ig gene promoters and enhancers, and evidence from over-expression and reporter assays confirmed Oct2-mediated, octamer-dependent gene expression. Complexity was added to the story when Oct1, an independently encoded protein, ubiquitously expressed from the *Pou2f1* gene, was characterized and found to bind to the octamer motif with almost identical specificity, and later, when the co-activator Obf1 (OCA-B, Bob.1), encoded by the *Pou2af1* gene, was cloned. Obf1 joins Oct2 (and Oct1) on the DNA of a subset of octamer motifs to enhance their transactivation strength. While these proteins variously carried the mantle of determinants of Ig gene expression in B cells for many years, such a role has not been borne out for them by characterization of mice lacking functional copies of the genes, either as single or as compound mutants. Instead, we and others have shown that Oct2 and Obf1 are required for B cells to mature fully *in vivo*, for B cells to respond to the T cell cytokines IL5 and IL4, and for B cells to produce IL6 normally during a T cell dependent immune response. We show here that Oct2 affects *Syk* gene expression, thus influencing B cell receptor signaling, and that Oct2 loss blocks *Slamf1* expression *in vivo* as a result of incomplete B cell maturation. Upon IL4 signaling, Stat6 up-regulates Obf1, indirectly via Xbp1, to enable plasma cell differentiation. Thus, Oct2 and Obf1 enable B cells to respond normally to antigen receptor signals, to express surface receptors that mediate physical interaction with T cells, or to produce and respond to cytokines that are critical drivers of B cell and T cell differentiation during a humoral immune response.

## Introduction

Octamer binding protein 2, or Oct2, is encoded by the *Pou2f2* gene. It was one of the first cell type-specific transcription factors identified and cloned (1). As indicated by its name, it is a founding member of a family of DNA binding proteins concurrently discovered, that share a conserved bipartite DNA binding domain comprising a homeobox-like domain and a second conserved sequence entitled the POU domain, for the *P*it1, *O*ct1/*O*ct2, *U*nc86 proteins (2). Oct2 binds to a conserved consensus DNA sequence, the “octamer motif” found in the promoters and enhancers of many genes, including those encoding immunoglobulins (3, 4). The Obf1 protein encoded by the *Pou2af1* gene, which is also known as OCA-B and Bob.1 was subsequently cloned using a yeast 1-hybrid screen for B cell proteins that physically interact with Oct1 or Oct2 (5–7). While Oct1/Oct2 and Obf1 share the capacity to bind to and activate genes adjacent to octamer motifs, they are selective in the genes to which they bind. The selectivity of target gene binding is determined, in part, by the sequence of the octamer motif, and whether it conforms to one of two classes of site, designated “PORE” and “MORE” motifs (8). Whether binding mediates activation or repression is also influenced by the participation of cofactors [reviewed by Tantin (9)], including Obf1, which can potentiate the transactivation potential of Oct1 and Oct2 (8, 10).

Oct2 is expressed primarily but not exclusively in the B cell lineage, where it increases with cellular activation (11). Neurons, macrophages, and T cells have also been shown to express *Oct2* (12–18). Oct2 is required for post-natal survival (19), so must regulate critically important genes outside of the immune system. These will not be discussed here. The *Oct2* gene is large, displays complex splicing patterns, and encodes protein isoforms with multiple essential activation domains (20–22). Oct2 is largely localized to the nucleus. *Obf1* expression is mostly restricted to B lineage cells, where it is also highly induced upon activation (23). Zwilling et al. (24) have reported expression in T cells, but myeloid cells do not express *Obf1* (15). A small protein of ~35 kDa, Obf1 is found in both the nucleus and cytoplasm, where a proportion may be tethered to the cell membrane after post-translational myristoylation (25), and a potential role for membrane-associated Obf1 in B cell receptor (BCR) signaling has been proposed (26).

A series of studies have shown that Oct2 and Obf1 are required for full functional and phenotypic maturation of B cells. In single knockout (KO) mice of each gene, peripheral B cells are numerically reduced and display some features of immature transitional cells (27, 28). The peritoneal B1 and splenic marginal zone (MZ) populations are missing in *Oct2^−/−^* mice (27, 29). *Obf1^−/−^* mice are viable and fertile, but show B cell developmental defects (30, 31), have an expanded B1 cell population (32). They also lack MZ B cells (33) and completely fail to produce germinal centers (GCs), the sites of cognate B cell:T cell interaction and expansion, upon immunization, or infection (34–37). Both Oct2- and Obf1-deficient splenic B cells display aberrant responses to BCR signaling and other characteristics of immature B cells (27, 34, 38). Oct2-deficient B cells also fail to respond to lipopolysaccharide (LPS), which signals through TLR4 (38). *In vivo*, serum immunoglobulin (Ig) levels in both mutants, particularly those that are T cell dependent, are strongly reduced (34–36, 38). Mice doubly deficient for Oct2 and Obf1 show a stronger humoral deficiency phenotype, reflecting the distinct activities of the two factors, but still express Ig genes (39). Thus, the two factors are not required, singly or in combination, for Ig gene expression by B cells.

Detailed functional studies on Oct2- and Obf1-deficient B cells *in vitro* and *in vivo* have identified a number of genes regulated by the two factors. Oct2 directly regulates the gene encoding CD36, a class B scavenger receptor family (40), but only in B cells, not in macrophages or dendritic cells (41–43). However, no role for CD36 in B cells has been determined (44). Oct2-deficent B cells have been shown to be defective in their responses to the T cell cytokine IL5 as a result of the direct regulation of the *Cd125* gene encoding the IL5Rα chain (29). IL5 promotes antibody-secreting cell (ASC) differentiation in mouse B cells (45), and *Oct2^−/−^* B cells are defective in this process (29). In another study, it was shown that both Oct2 and Obf1 contribute to the regulation of IL6 production by activated B cells, through direct effects, at least by Oct2, on the *Il6* gene (11). As Obf1 does not contact DNA (5), it is difficult using current procedures to prove direct interaction of Obf1 with putative target gene loci. IL6 is important during T follicular helper (Tfh) cell polarization (46). We have also shown, using the same quantitative tools that identified the role of Oct2 and IL5 in ASC differentiation, that Obf1 is required for T cell dependent ASC differentiation, but not isotype switching, both *in vitro* and *in vivo* (29).

In addition to these established roles for Oct2 and Obf1 in B cells, we include below data from studies on other genes that we have found to be differentially regulated in Oct2- and Obf1-deficient B cells. These include the genes encoding the Syk protein, which is an important transducer of BCR signals and Slamf1, an essential mediator of cell:cell contact, especially in the context of a developing GC. Expression of Syk and Slamf1 are sensitive to Oct2 loss, through different mechanisms. We also show that Obf1 is downstream of Stat6 in the IL4 signaling pathway of B cells, with Xbp1, another Stat6 target, its direct activator. We include these data to add to our understanding of the valuable roles that Oct2 and Obf1 play in B cell responses to antigen and to T cell help.

## Results

**Figure 1:**
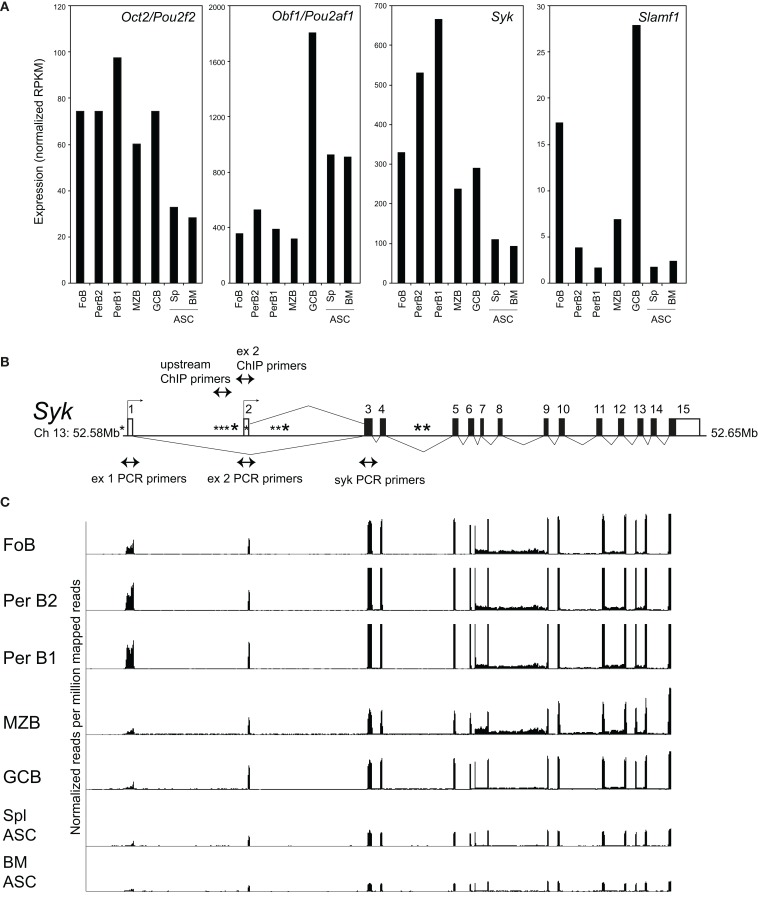
**Expression of Oct2, Obf1, Syk, and Slamf1 in peripheral B cell populations**. **(A)** RNAseq data measuring expression of *Oct2/Pou2f2, Obf1/Pou2af1, Syk*, and *Slamf1* in B cell populations sorted *ex vivo* from naïve C57BL/6 mice. FoB, follicular B cells from spleen (small B220^+^, IgM^+^, IgD^+^) PerB1 and PerB2, B220^+^ cells from peritoneal lavages of naïve mice, stained with CD23 and Mac1. B1 cells were CD23*^−^* and Mac1^lo^ and B2 cells were CD23^+^ and Mac1*^−^*; MZB, splenic marginal zone B cells, B220^+^, IgM^hi^, CD21^hi^; GCB, germinal center B cells (B220^+^, Fas^+^, GL7^+^) from spleens of mice immunized 8 days previously with SRBC; ASC, antibody-secreting cells sorted as syndecan1^+^, GFP^+^ cells from spleens (Spl), and bone marrows (BM) of mice carrying the *Blimp-GFP* reporter gene (47). Data were derived from at least two independent biological replicates in all cases. Because Ig sequences can represent >70% of the RNA from plasma cells (data not shown), the RNAseq data shown in the figure excludes all reads mapping to the Ig (heavy and light chain) loci as described in Section “Materials and Methods.” **(B)** Structure of the mouse *Syk* gene, showing exons, alternative transcriptional start sites (small arrows), the locations of a perfect consensus octamer motif (*) and the positions of PCR primers used here. Filled boxes indicate protein coding sequence, and open boxes, sequence comprising the 5′ and 3′ untranslated regions of *Syk* mRNA. **(C)** RNAseq tracks showing expression of the *Syk* gene exons in different sorted B cell populations, normalized to library size, and aligned with the gene structure of **(B)**. Note that exon 1, as shown in this panel, is not included in the RefSeq (Mouse mm9, July 2007) map of *Syk* mRNA, but is represented in alternate *Syk* transcripts ENSMUST00000120135 and ENSMUST00000118756 in the Ensembl database.

Consistent with their distinct roles *in vivo*, Oct2 and Obf1 have quite distinct patterns of expression in peripheral B cells, as measured by RNAseq of sorted populations from naive C57BL/6 mice (Figure 1A). Oct2 levels are highest in B1 cells of the peritoneal cavity, and decline with terminal differentiation to ASCs. In contrast, Obf1 levels peak in GC B cells, which require Obf1 for their generation, and remain high in ASC. For contrast, expression of *Syk* and *Slamf1* in the same populations are shown in Figure 1A, as these two genes are influenced directly and indirectly, respectively, by Oct2, and will be discussed below.

### Oct2 modulates B cell receptor signaling by fine-tuning Syk expression

A microarray screen for Oct2-dependent genes identified the tyrosine kinase *Syk* as a potential target gene. Characterization of the murine *Syk* promoter using 5′ RACE identified two alternative transcriptional initiation sites (Figure 1B) that append alternative 5′ non-coding exons to *Syk* mRNAs in B cells. Both transcripts encode the same protein, as the start of Syk translation lies in an exon common to the two transcripts. RNAseq data for *Syk* in sorted B cell populations show that the usage of exons 1 or 2 varies subtly among them (Figure 1C).

**Figure 2:**
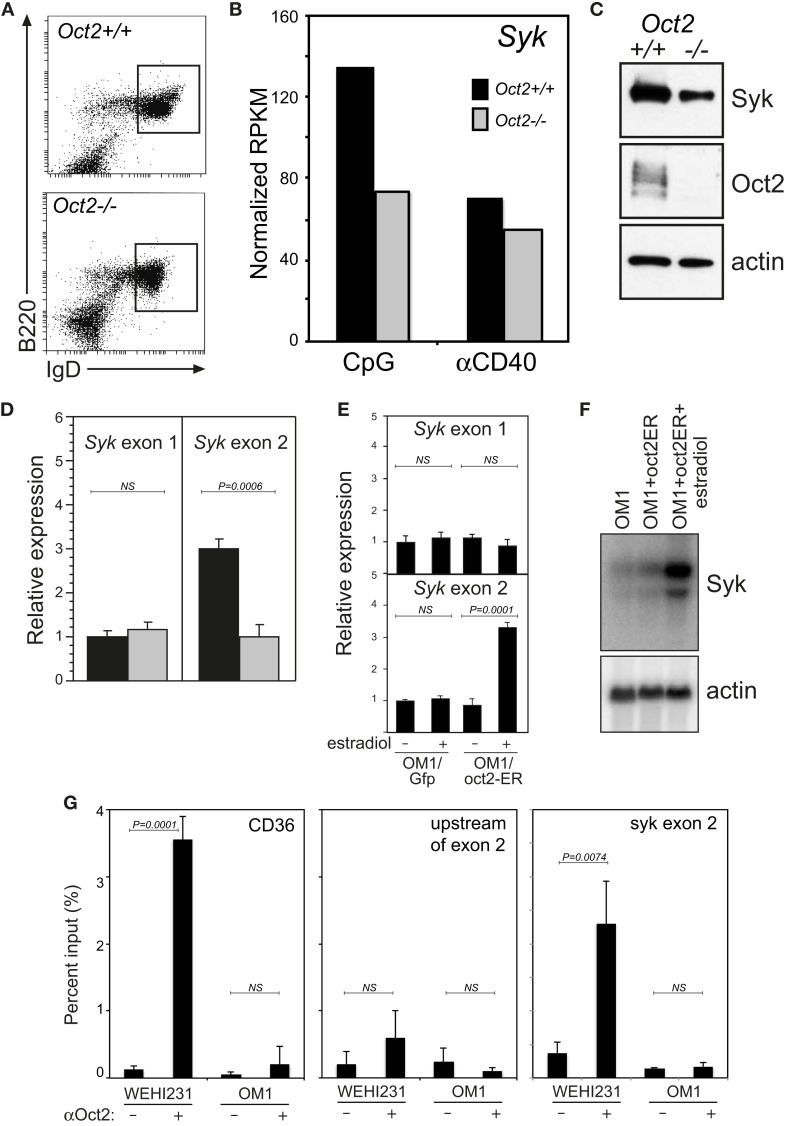
**Oct2 directly and selectively activates transcription from Syk exon 2**. **(A)** Splenic B cells from WT and Oct2 KO mice stained for B220 and IgD expression and sorted for phenotypically mature Fo B cells. **(B)** *Syk* RNAseq data from WT (black bars) and *Oct2^−/−^* (gray bars) B cells, sorted as in **(A)**, activated for 48 h with CpG or anti-CD40. **(C)** Syk protein in sorted resting Fo B cells from *Oct2*^+^*^/^*^+^ or *Oct2^−/−^*mice. **(D)** qPCR of *Syk* mRNA distinguishing transcripts initiated at exons 1 or 2 in sorted splenic Fo B from WT and Oct2 KO mice. Expression is relative to that of the *hmbs* housekeeping gene. Values are means ± SD of triplicate assays. **(E)** Specific induction of transcription from *Syk* exon 2 upon Oct2 over-expression in OM1 cells, which are *Oct2^−/−^* (48), as shown by qPCR. Values are means ± SD of triplicates. **(F)** Northern blot for total *Syk* mRNA from a parallel experiment. **(G)** Quantitation of Oct2 chromatin immunoprecipitation (ChIP) qPCR data, showing enrichment of the *Cd36* promoter [a known Oct2 target gene (41, 42)] and *Syk* sequences upstream of exon 2. Oct2:DNA complexes were precipitated from WEHI231 B lymphoma cells (which are *Oct2*^+^*^/^*^+^) and OM1 B lymphoma cells. Oct2 does not bind appreciatively to adjacent sequences in *Syk* intron 1/2. Values are means ± SD of triplicate assays. *P* values were calculated using the unpaired Student’s *t*-test; NS, not significant.

The promoter upstream of exon 2 is positively regulated by Oct2. B cells sorted from *Oct2^−/−^* mice (Figure 2A) have a lower level of *Syk* transcripts and protein than wild type (WT) mice (Figures 2B,C). Using qPCR to distinguish *Syk* transcripts derived from exon 1 or exon 2, we found that those derived from exon 2 were selectively reduced in *Oct2*^−/−^ B cells (Figure 2D). To confirm the influence of Oct2 on the Syk exon 2 promoter, we stably introduced an estradiol-inducible form of Oct2 (29) into a cloned *Oct2^−/−^* B lymphoma cell line, OM1 (48). Upon treatment with estradiol, there was no effect on expression from exon 1 with either the vector only control or the inducible Oct2 construct. However, estradiol induction of Oct2 selectively enhanced *Syk* expression from exon 2 and culminated in markedly increased *Syk* mRNA levels (Figures 2E,F). A DNA sequence search revealed three perfect consensus octamer sequences in the *Syk* gene (Figure 1B). Chromatin immunoprecipitation (ChIP) of Oct2 on DNA from the WT B lymphoma WEHI231 showed strong enrichment of DNA from the promoter of the known Oct2 target gene *Cd36* (Figure 2G). The DNA adjacent to the perfect octamer sequence upstream of exon 2 was also significantly enriched in the ChIP, but adjacent sequences in intron 1/2 were not. Thus, Oct2 can directly increase *Syk* levels in B cells, acting at one of two alternative promoters.

**Figure 3:**
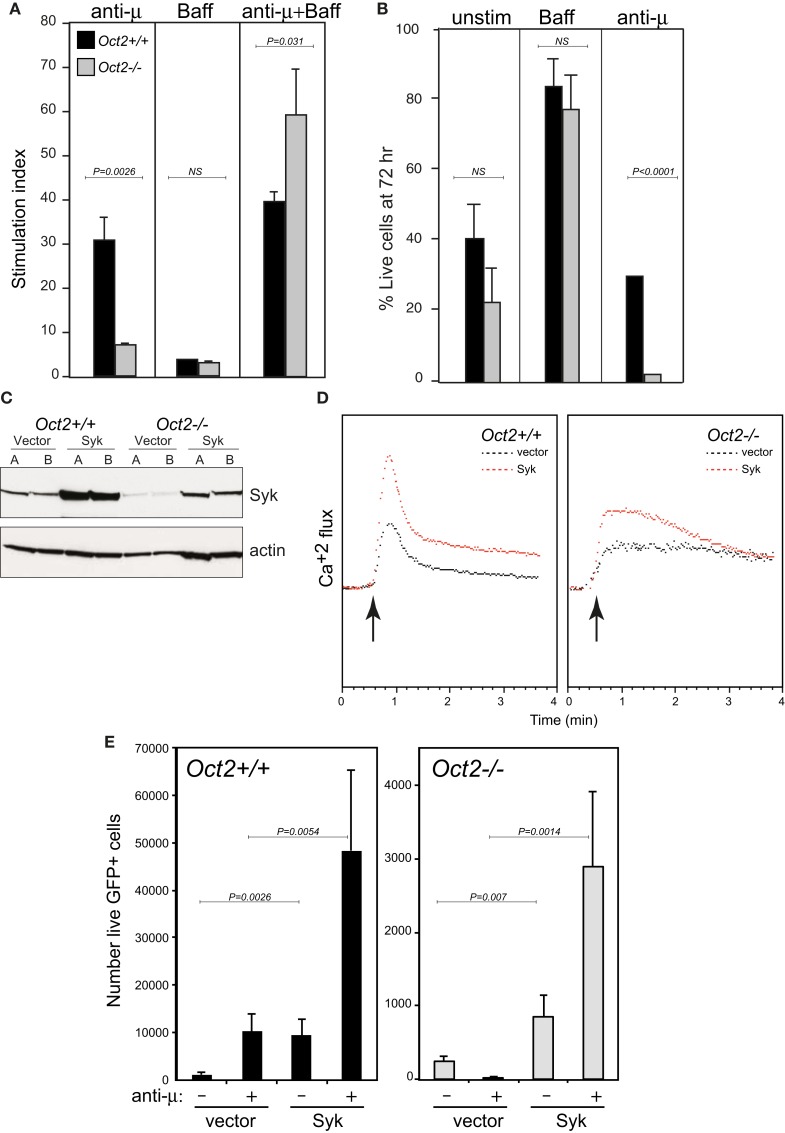
**Oct2 is required for normal signaling from the B cell receptor, and ectopic Syk expression enhances the response in both WT and KO B cells**. **(A)** B cell proliferation in response to BCR signaling, Baff, or both in combination after 3 days. Stimulation index is calculated as proliferation relative to unstimulated cells. Filled bars, *Oct2*^+^*^/^*^+^ gray bars, *Oct2^−/−^*. All values are the mean of triplicates ± SD. **(B)** Survival, assessed by propidium iodide exclusion, of B cells cultured with Baff or with anti-μ for 3 days. Filled bars, *Oct2*^+^*^/^*^+^ gray bars, *Oct2^−/−^*. All values are the mean of triplicates ± SD. **(C)** Syk protein levels in cloned B lymphoma cells transduced with a Syk-expressing retrovirus. The *Oct2*^+^*^/^*^+^ and *Oct2^−/−^*cell lines are BC1 and OM1, respectively (48). **(D)** Cytometric measurement of Ca2^+^ flux in clones of BC1 and OM1 cells transduced with vector only (black) or a Syk-expressing retrovirus (red). The arrow indicates the timing of addition of anti-μ to cross-link the BCR. **(E)** Primary splenic B cells from *Oct2*^+^*^/^*^+^ and *Oct2^−/−^*mice were activated, transduced (see Materials and Methods) and subsequently treated with anti-μ. The number of live, transduced (GFP^+^) cells in each culture after 48 h is shown. Values are means (*n* = 4) ± SD. Filled bars, *Oct2*^+^*^/^*^+^ gray bars, *Oct2^−/−^*.

As mentioned above, Oct2 is required for the full functional and phenotypic maturation of B cells, such that peripheral B cells in *Oct2^−/−^* mice are numerically reduced and display some features of immature transitional cells, and the B1 and MZ populations are missing (27, 29, 38). Like transitional B cells, Oct2-deficient B cells are killed rather that activated by BCR cross-linking [Ref. (38, 49); see also Figure 3A]. However, *Oct2^−/−^* B cells respond normally to the survival factor Baff/BlyS, and survive to expand normally when BCR signaling occurs in the presence of Baff (Figures 3A,B and data not shown). *Oct2^−/−^* mice expressing a Bcl2 transgene still lack B1 and MZ B cells (data not shown). Thus abnormal survival properties are not responsible for the lack of these two populations in the Oct2 mutant mice.

We speculated that the reduced Syk levels in *Oct2^−/−^* B cells might contribute to their failure to mature *in vivo* and respond to BCR signals *in vitro*. We constructed a retroviral vector expressing Syk, and infected and cloned WT (BC1) and Oct2-deficient (OM1) lymphoma cells (Figure 3C). Boosting Syk protein levels enhanced the BCR response, as measured by calcium flux, in both WT and *Oct2^−/−^* cells (Figure 3D). Finally, using transduction of primary B cells (see Materials and Methods), we found that elevating Syk levels improved the proliferation of WT B cells, both unstimulated, and more strongly, upon BCR cross-linking, and that *Oct2^−/−^* B cells complemented with Syk retrovirus were activated to expand, rather than be killed by a BCR signal (Figure 3E). The rescue was not complete, as cell survival was still lower overall in the mutant cell cultures. This is likely to reflect technical limitations of the assay, including comparative infectivity of WT and mutant cells, and the correct timing of exogenous *Syk* expression in the context of the BCR signal. However, the results strongly suggest that Syk levels are limiting in *Oct2^−/−^* and, to a lesser extent, in WT B cells. We propose that Oct2 regulates *Syk* gene expression to enable positive selection through the BCR and therefore entrance to the mature follicular B cell pool, and it may similarly enable differentiation of B1 and MZ B cells, which are highly dependent on BCR signal strength.

### Oct2 indirectly and selectively regulates Slamf1 expression on B cells

**Figure 4:**
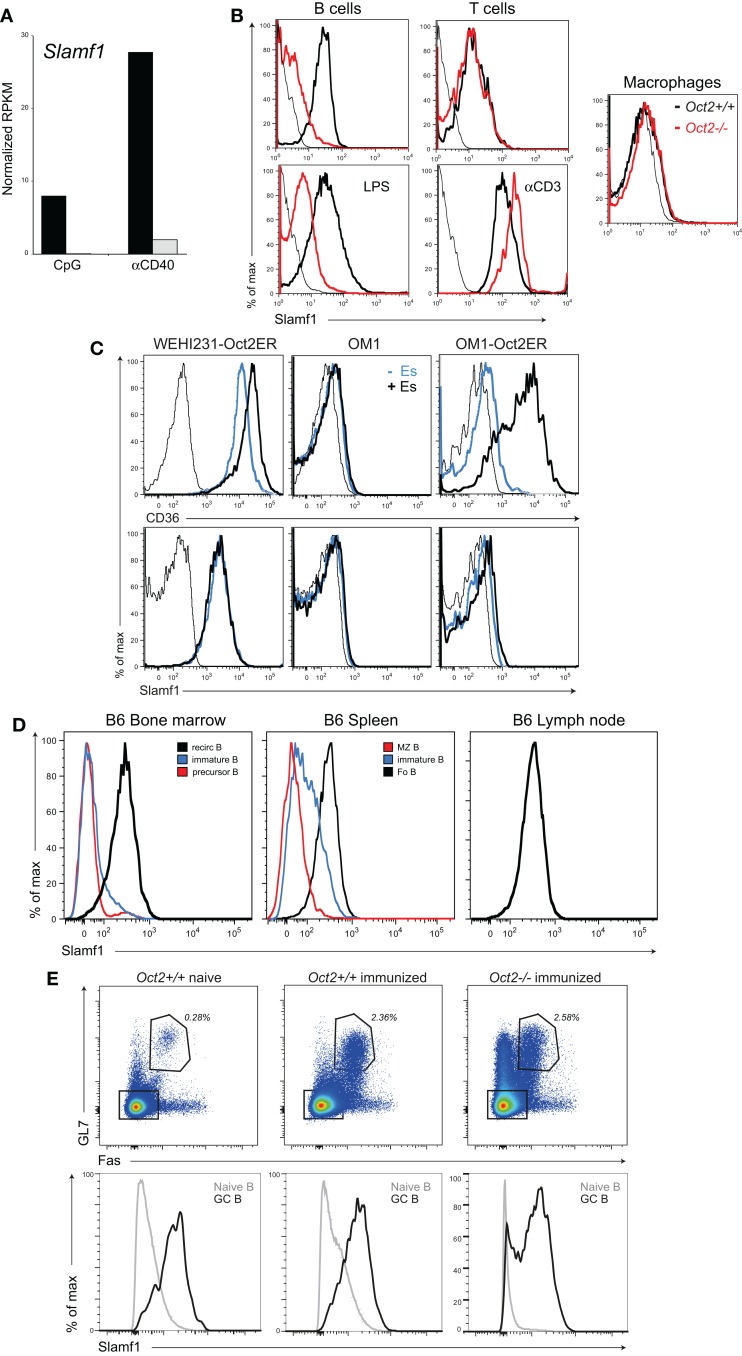
**Oct2 influences and Slamf1 expression in B cells**. **(A)** Analysis of Slamf1 expression in Oct2-null and *Oct2*^+^*^/^*^+^ B cells activated *in vitro*. Cells were cultured for 48 h in the presence of CpG or aCD40 before RNA was prepared for RNA sequencing. Filled bars, *Oct2*^+^*^/^*^+^ gray bars, *Oct2^−/−^*. **(B)** Slamf1/CD150 protein expression in cells of the indicated genotypes. Cells were assessed directly *ex vivo* (resting, top panels), or were activated *in vitro* for 48 h with either LPS (B cells) or anti-CD3 (T cells). Macrophages were expanded from fetal liver as described in Section “Materials and Methods.” In the histograms, WT cells are represented with heavy black lines and *Oct2^−/−^* as red. Unstained controls are indicated by thin black lines. **(C)** WEHI231 cells (*Oct2*^+^*^/^*^+^) and OM1 cells (*Oct2^−/−^*), either uninfected or transduced with an Oct-ER expression vector, were cultured in the presence or absence of estradiol (Es) for 24 h and CD36 and Slamf1 levels were determined by flow cytometry. Thin lines indicate background fluorescence of unstained controls, blue lines represent CD36 and Slamf1 levels in uninduced cultures, and heavy black lines, the levels after Es induction. **(D)** Slamf1 expression during B cell maturation. Colors indicate the populations represented in each histogram. For bone marrow, recirculating B cells were B220^++^ and IgM^+^, immature B were B220^+^ and IgM^++^, and precursor B were B220^+^ and IgM*^−^*. For spleen, immature B cells were IgM^hi^, IgD^lo^, MZ B cells were IgM^hi^, CD21^hi^, and Fo B cells were IgM^lo^, IgD^hi^, CD23^+^, and CD21^+^. Lymph node B cells were IgM^lo^, IgD^hi^, and CD23^+^. **(E)** Flow cytometric analysis of splenic B cells from naïve and immunized mice (9 days after SRBC immunization). Top panels show the percentage of GL7^+^Fas^+^ GC B cells (gated) among total B220^+^ cells in spleens from mice reconstituted with WT or *Oct2^−/−^* fetal liver. Bottom panels show the Slamf1 levels on non-GC (gray lines) and GC (black line) B cells for each animal, gated as shown in the upper panels.

*Slamf1* encodes CD150, a lymphocyte signaling and adhesion molecule (50) that is expressed in B cells and T cells and at very low levels in myeloid cells (see www.immgen.org). Our RNAseq analysis of activated B cells indicated that *Slamf1* was expressed at abnormally low levels in Oct2 KO B compared to controls (Figure 4A). This was confirmed by flow cytometric analysis (Figure 4B). Resting WT and LPS-activated B cells expressed similar levels of Slamf1, but levels on Oct2-deficient B cells were much lower than controls under both conditions. Activated WT T cells up-regulated Slamf1 from resting levels. However, Slamf1 was not Oct2-dependent in resting or activated T cells, or in macrophages expanded from fetal liver, despite Oct2 normally being expressed in these cell types (15, 16, 18).

We next asked whether Slamf1 was a direct Oct2 target using cloned WT (WEHI231) and KO (OM1) B lymphoma lines transduced with the inducible Oct2-ER vector, as described above. As expected, the Oct2 target *Cd36* gene was expressed in WEHI231 but not OM1 (Figure 4C). Estradiol treatment enhanced *Cd36* levels in WEHI231, and strongly induced *Cd36* expression in OM1 cells. However, *Slamf1* expression, while low in the *Oct2^−/−^* line, was not increased by Oct2 induction. Therefore, Oct2 is unlikely to directly regulate *Slamf1* transcription in B cells.

Examination of the pattern of expression of the *Slamf1* gene during B cell development, using the Immgen database (http://www.immgen.org) and by flow cytometry of bone marrow (BM) and peripheral B cell populations indicated that Slamf1 is a marker of B cell maturation, appearing during the transition from immature (IgM^hi^/IgD^lo^) to mature (IgM^lo^/IgD^hi^) follicular B cells of the spleen (Figure 4D), with MZ B cells expressing intermediate Slamf1 levels. We conclude that loss of Oct2 blocks B cell maturation before the Slamf1^+^ stage, thereby indirectly regulating its expression.

It has been shown that B cells lacking Slamf1 cannot form the lasting interactions with Tfh cells that are required for GC formation (51), and yet Oct2 mice do form GC upon infection and immunization (11). We immunized WT and KO mice with SRBC and stained GC cells for Slamf1 9 days later. *Oct2*^+^*^/^*^+^ and *Oct2^−/−^*GC B cells expressed similar levels of Slamf1. This indicates that Oct2 is dispensable for *Slamf1* expression, and that endogenous signals driving the GC response override the Oct2 maturation defect *in vivo* (Figure 4E). To explore the nature of these signals, we tested the capacity of a number of mitogens and cytokines to induce Slamf1 expression on Oct2-null B cells *in vitro*. Anti-μ, anti-CD40, Baff, and IL4, tested singly or in all possible combinations, failed to induce appreciable Slamf1 on *Oct2^−/−^* B cells, suggesting that other factors and cells contribute *in vivo*. Collectively these studies point to an Oct2-regulated differentiation step that enables efficient FoB cell maturation.

### Obf1 enables T cell dependent ASC differentiation driven by IL4

We have shown that both Oct2 and Obf1 affect a B cell’s capacity to differentiate to ASC in response to particular cytokines, with Oct2 regulating the response to IL5 (29), and Obf1 being essential for ASC differentiation driven by IL4 (52).

**Figure 5:**
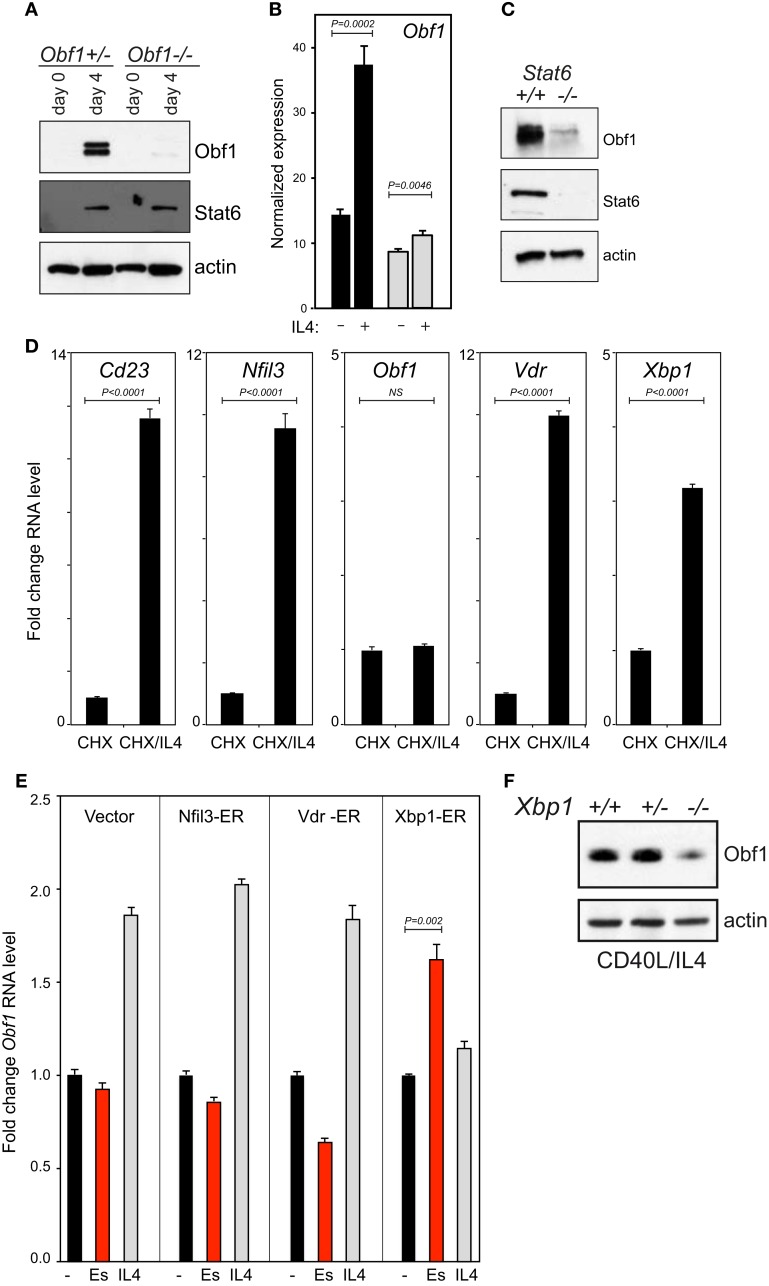
**Obf1 is downstream of Stat6 in the IL4 signaling cascade**. **(A)** Western blots to detect Obf1 and Stat6 proteins in purified B cells of the indicated genotypes, harvested 48 h after anti-CD40 ± IL4 treatment. Actin is included as a loading control. **(B)** RNA sequencing data showing *Obf1* expression 48 h after anti-CD40 ± IL4 treatment in WT (black bars) and *Stat6^−/−^* B cells (gray bars). **(C)** Western blot on the same samples as in **(B)**, to detect Stat6 and Obf1 proteins. **(D)** Induction of IL4 responsive genes in the presence of the translational inhibitor cycloheximide (CHX), as measured by qPCR. **(E)** Induction of *Obf1* RNA levels in B lymphoma cell lines each expressing a ER fusion of each of the transcriptional regulators that are direct IL4 target genes: *Nfil3, Vdr*, and *Xbp1*. Cells were untreated or treated with either estradiol or IL4 for 24 h. In **(B,D,E)**, values are means ± SD for triplicate samples. **(F)** Western blot to detect *Obf1* protein in purified B cells from *Xbp1*^+^*^/^*^+^, heterozygous, or conditional KO mice. Cells were cultured in anti-CD40 plus IL4 for 48 h before analysis. In **(B,D,E)**, means ± SD for triplicate measurements are shown, with *P* values determined using the unpaired Student’s *t*-test. NS, not significant.

We determined that Obf1 lies downstream of Stat6 in the IL4 signaling cascade (Figures 5A–C). Interestingly, while the IL4/Stat6 axis drives both isotype switching and ASC differentiation (45), Obf1 is dispensable for IL4 driven switching (52). In order to learn how IL4 and Stat6 regulate *Obf1* expression, we performed microarray analysis on the WEHI231 B lymphoma, an IL4 responsive line. Because Stat6 exists in a latent form in the cytoplasm, and is activated by phosphorylation to enter the nucleus and act as a transcription factor (53), direct Stat6 targets would be activated after IL4 signaling even in the absence of new protein synthesis. Cells therefore were treated with cyclohexamide (CHX) to inhibit translation, and 1 h later, IL4 was added. A parallel culture did not receive IL4. After another 4 h, RNA was prepared from both. Analysis using Illumina Sentrix Mouse v1.1 arrays identified a small number of genes whose expression rose two to fourfold during this short period of IL4 stimulation, genes likely to be regulated directly by Stat6. Among these were three transcriptions factors: Nfil3, Vdr, encoding the 1,25-dihydroxyvitamin D3 receptor, and Xbp1. Their induction under these conditions was validated by qPCR (Figure 5D). Interestingly, *Obf1* transcription was not elevated by IL4 treatment in the presence of CHX, indicating that it is not a direct Stat6 target gene (Figure 5D).

As *Obf1* RNA is elevated by IL4 treatment in B cells in the absence of CHX (Figure 5B), we hypothesized that one of the three transcription factors directly regulated by Stat6 might drive *Obf1* expression. We therefore constructed ER fusion vectors for each to determine their effects on Obf1 expression. The Xbp1-ER expression vector contained the mature, processed, and active form of this factor (54). Clones of each line were cultured for 24 h unstimulated, with estradiol to induce each fusion protein, or with IL4. IL4 caused an increase in *Obf1* RNA levels in all cases, as expected. However, estradiol induction only increased *Obf1* levels significantly in the Xbp-ER expressing cell line (Figure 5E). Finally, primary splenic B cells from *Xbp1*^+^*^/^*^+^, heterozygous and conditional KO mice were stimulated for 48 h with CD40 ligand and IL4, and protein extracts prepared. Western blots showed that Xbp1-null B cells express markedly less Obf1 than the controls (Figure 5F). These data indicate that Nfil3 and Vdr do not influence *Obf1* expression, but that Xbp1 has the capacity to directly activate the *Obf1* gene in response to IL4/Stat6 signaling in B cells.

## Discussion

**Figure 6:**
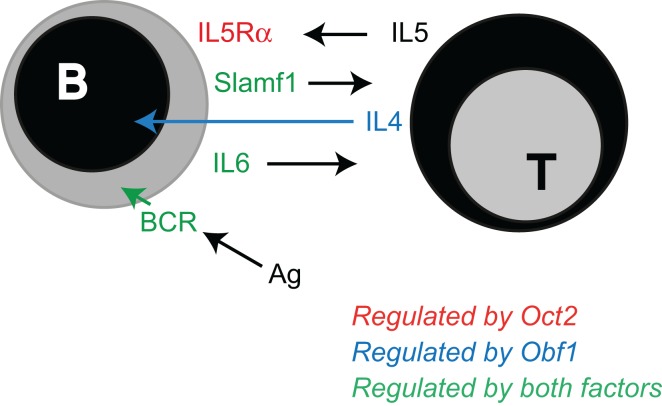
**Oct2 and Obf1: finessing the B cell**. Summary of published work and data discussed here, identifying the B cell responses that depend upon Oct2 and Obf1 for their optimal function. Oct2 (red arrow) selectively enables B cells to respond to IL5, a T cell-derived driver of ASC differentiation (45). Obf1 (blue arrow) selectively mediates IL4 driven ASC differentiation, downstream of Stat6. Both Oct2 and Obf1 (green arrows) enable BCR signaling, B cell maturation and indirectly, expression of Slamf1, an important mediator of B cell:T cell interactions during a humoral immune response.

The data we present here adds to a growing view of Oct2 and Obf1 as essential contributors to the sensing capacity of B cells (Figure 6). These two factors enhance the cell’s ability to deliver a BCR signal to drive maturation, or to sense a foreign antigen and become activated. We show here that Oct2 may do so by fine-tuning Syk levels. Since Oct2 loss blocks peripheral B cell maturation, such that MZ B cells are missing and mature FoB reduced, Oct2 may play its most important role prior to a divergence point of Fo and MZ B cells (55). The immature Slamf1*^−^*, BaffR^+^, CD23^+^, CD21^+^, IgM^hi^, D^lo^, HSA^hi^ phenotype of *Oct2^−/−^* B cells does not neatly fit into the phenotypic transition of immature to mature B cells (55), and may represent a normally transient phase of B cell maturation. Obf1 is required for normal B cell maturation and MZ B cell development (33), but as Obf1 does not influence *Syk* expression, it is likely that the two factors act through unique subsets of target genes for this aspect of B cell development. Indeed, it has been reported that Obf1 positively regulates *SpiB* gene expression (56), and that SpiB is required for normal B cell maturation and BCR signaling, through regulation of *c-rel* (57, 58).

T cell dependent antibody responses depend upon Oct2 and Obf1 in several ways. Both Oct2 and Obf1 are required for B cells to produce normal levels of IL6, a cytokine important in Tfh maturation in the context of a Slamf1-mediated B cell:Tfh cell interaction (11, 59, 60). These molecular interactions enable the initiation of a fruitful T cell-dependent humoral immune response. For example, IL6 produced by activated B cells early in the GC response reinforces the early dendritic cell signal that initiates Tfh differentiation, and IL21 produced by the nascent Tfh enhances both Tfh function and B cell differentiation into GC B cells and ASC (61–66). Slamf1, which is indirectly dependent upon Oct2, is required to prolong the B:Tfh interaction while these important signals are exchanged. Subsequently, Oct2 and Obf1 enable B cells to respond to other Th cell cytokines that drive ASC differentiation. Oct2 regulates *Cd125* expression (29), and so expression of the high affinity receptor for IL5, an ASC differentiation factor (45). *Obf1* is required for a normal B cell response to IL4, a growth, survival, isotype switching and differentiation factor for B and plasma cells [(45, 67) and this study].

Except for the specific case of Obf1 and a subset of V_L_ genes (68), there is no evidence that Oct2 or Obf1 are required for Ig gene expression or for antibody secretion in ASC generated from single or double KO mice (29, 39, 52). Interestingly, however, we show here that Xbp1, which is highly expressed in ASC and known to be required for ASC function [(69–72) and our unpublished data], can directly activate *Obf1*. Shen and Hendershot (73) have also shown in ASC that *Obf1* is a direct target of Xbp1. Accordingly, *Obf1* expression is elevated in normal ASC compared to B cells, unlike Oct2 expression, which declines with differentiation (Figure 1). Xbp1, normally associated with the unfolded protein response in ASC, is an IL4 response gene in B cells. We confirm here that *Obf1* is an Xbp1 target gene in FoB cells, and that IL4 and Stat6 directly induce Xbp1. Iwakoshi et al. (70) have earlier shown that, in the context of ASC differentiation, IL4 strongly induces *Xbp1* expression and Stat6 is required. Thus, the selective ASC differentiation defect in Obf1 null B cells under T cell-dependent conditions may reflect an important Stat6-Xbp1-Obf1 axis.

Instead of direct influences on Ig gene expression, poor humoral immune responses in Oct2 or Obf1 mutant mice more likely reflects a paucity of differentiated peripheral B cell populations and weak to absent influences of T cells on these B cells. *Oct2* and *Obf1* are most highly expressed in GC B cells, with Obf1 being essential for their differentiation, but Oct2 dispensable (11). In both mutants, the B cells defects are cell intrinsic. Outstanding questions remain: what are the genes that Oct2 and Obf1 regulate to ensure full peripheral maturation and competent BCR signaling? What are the Obf1 regulated genes that are essential for GC development under all circumstances tested so far, including immunization, infection, and autoimmunity (11, 32, 37)? While these critical Obf1 target(s) are not yet known, candidates such as Bcl6 (or its co-repressor MTA3), Bach2, Irf4 and CXCR5, all critical for normal GC formation or maintenance [see Ref. (74)], can be excluded, as they are not influenced by Obf1 loss (our unpublished data). Ongoing genome-wide RNA expression analysis, coupled with ChIPseq, will enable detailed characterization of the full Oct2 and Obf1 gene regulatory networks, and their shared and unique responsibilities in delivering effective B cell immunity.

## Materials and Methods

### Cell lines, cell culture, and retroviral transduction

B lymphoma cell lines used here were all generated in house: WEHI231 (75), BC1, and OM1 (48). Primary splenic B and T cells were purified using anti-B220 or anti-CD4 microbeads (Miltenyi), as described (11). *Oct2*^+^*^/^*^+^ and *Oct2^−/−^*macrophages were expanded *in vitro* from E13 fetal liver using M-CSF, as described (15). Retroviral transduction in all cases used the pMX-pie vector and was preformed using spin infection as described (48, 76). For lymphoma lines, cells were cloned post-infection as single GFP^+^ cells in puromycin-supplemented medium.

For the retroviral complementation experiment of Figure 3E, primary splenic B220^+^ cells were stimulated for 24 h with CpG [1 μM oligonucleotide CpG 1668 (sequence 5′-TCCATGA CGTTCCTGATGCT-3′), fully phosphothioated GeneWorks] to promote cell cycling and enable retroviral infection. After overnight culture, cells were washed and resuspended in medium at 0.5 × 10^6^ cells/ml, without CpG but containing anti-μ [10 μg/ml AffiniPure F(ab′)_2_ fragment, goat anti mouse Jackson Laboratories] and/or Baff (250 ng/ml a kind gift from Jürg Tschopp). Cell survival in transduced (GFP^+^) cells was assessed after a further 48 h by flow cytometry, propidium iodide exclusion, and cell counting using internal microbead controls.

Retroviruses expressing transcription factors fused to the human estrogen receptor (hER) dimerization domain were generated by amplification of each factor’s ORF, and sequencing each amplified product to ensure it was mutation-free and in frame with the hER. Production of ER fusion proteins of the correct size was confirmed by western blots of infected or transfected cells. Anti-CD40 (clone FGK4.5) was prepared in house and used at 10 μg/ml. β-Estradiol (Sigma) was used at 10 μM, cycloheximide at 50 μM.

### Mice

*Oct2^−/−^, Obf1^−/−^*, and *Stat6^−/−^* mice have been described previously (19, 36) *Xbp1^fl/fl^Cd19^Cre/^*^+^mice, where *Xbp1* is conditionally deleted in the B cell lineage, were generated by Hetz et al. (77) and further described in Taubenheim et al. (72). All mice were maintained on a C57BL/6 background, and all experiments conformed to the relevant regulatory standards of the Animal Ethics Committee of the Walter and Eliza Hall Institute of Medical Research (AEC Projects 2010.010 and 2013.014).

### Antibodies

For westerns, antibodies used were specific for Oct2 (clone 9A2, in house), Obf1 (clone 6F10, in house) Stat6 (S-20), Syk (N-19), and actin (I-19) all from Santa Cruz and Xbp1 (Ab37152-100, Abcam). For flow cytometry, antibodies used were specific for B220 (RA3-6B2), IgD (11-26c.2a), fas/CD95 (Jo2), all from BD Pharmingen Slamf1/CD150 (TC15-12 F12.2, Biolegend), and GL7 (eBioscience). Calcium flux was assessed cytometrically as described (78).

### Microarrays

Illumina Sentrix Mouse v1.1 arrays were probed with RNA prepared from independent B lymphoma cell lines: to identify Oct2-dependent genes, two independent clones of OM1 cells stably transduced with a control vector or one expressing an estradiol-inducible Oct-ER fusion protein were treated *in vitro* for 6 or 48 h with estradiol prior to RNA preparation. For Obf1 targets, two clones of the *Obf1^−/−^* BM1 lymphoma line (78), transduced with vector control or an Obf1-ER expression vector, were induced in a similar manner and RNA prepared and analyzed.

### RNA sequencing

Peripheral B cell populations were sorted from naïve or immunized C57Bl/6 mice as described in the legend to Figure 1. Two independent biological replicates were prepared for each population, except for spleen and BM ASC. Because of the paucity of ASC in these tissues, cells were pooled from three to four individuals before sorting. Two such pools were processed independently for sequencing. Normalized expression levels are shown in the graphs. As more than 70% of reads in RNA from ASC map to the Ig loci, all Ig reads were excluded from the data, for all populations, before normalization to generate the values shown in Figure 1A. Specifically, all reads from the IgH locus on chromosome 12, NC_000078.6 (positions 113,258,768–116,009,954), all reads from the Igλ locus on chromosome 16, NC_000082.6 (positions 19,026,858–19,260,844), and all reads from the Igκ locus on chromosome 6, NC_000072.6 (positions 67,555,636–70,726,754) were excluded. For Figure 2B, *Oct2*^+^*^/^*^+^ and *Oct2^−/−^*B cells were sorted, as in Figure 2A, from spleen of two independent mice of each genotype and activated for 48 h before RNA extraction and preparation for sequencing. As these conditions do not induce ASC differentiation, Ig sequence reads were not excluded from analysis of these samples.

### Primers

The starts of *Syk* transcription in primary B cells were determined directly using FirstChoice^®^ RLM-RACE (Ambion) and nested *Syk* gene specific primers specific for the first Syk coding exon: 5′-GTAGGTCAGGTGGTTGGCGCTGTCCACAGC-3′ and 5′-CCCGCCATGTCTGCACCCCTTCAGAGTTC-3′.

For exon-specific *Syk* qPCR, these primers were used:

5′*Syk* exon 1: 5′-CAGTGACTGCGGCTGAGCGCGGACC-3′
5′*Syk* exon 2: 5′-CAGCAGGAAACCTCCACTTGCTCTCC-3′
Common 3′ *Syk* primer: 5′-CCATGTCTGCACCCCTTCAGAGTTC-3′.

For *Syk* ChIP PCR, these primers were used:

Exon 2: fwd 5′-GCCTAGGCCACGATGGTCAAAGGAGG-3′ and
rev 5′-GGAGAGCAAGTGGAGGTTTCCTGCTG-3′.
Upstream of exon 2: fwd 5′-CCATTGGTGGGCCCTCAGCTTGGTTC-3′ and
rev 5′-GACCAGAGAAGAAATGGCCTCAGAAGACAGG-3′.

Chromatin immunoprecipitation was conducted as previously described (29), using high titer, polyclonal anti-Oct2 rabbit serum generated in house.

Primers for other qPCR were:

*Stat6:* fwd 5′-CTGCTGGGCCGAGGCTTCACATTT-3′
rev 5′-TCAGGGGCCATTCCAAGATCATAAGGT-3′
*CD36*: fwd 5′-GGAGGCATTCTCATGCCAGTCGGAGAC-3′
rev 5′-CAAAACTGTCTGTACACAGTGGTGCCTG-3′
*Slamf1*: fwd 5′-GGGAGCTATCCAGATCACCTG-3′
rev 5′-CGTTCTCCTCCACGCTCAC-3′
*Cd23*: fwd 5′-GACACTGCAATTCAGAATGTCTCTCATG-3′
rev 5′-GCTTCTGTTCAGCTTGGAGTTCTTGCAAG-3′
*Nflil3*: fwd 5′-CTCACGGACCAGGGAGCAGAACCACG-3′
rev 5′-CAGGTCTTAAGGACTTCAGCCTCTCATCC-3′
*Obf1*: fwd 5′-CGGTGTTGACCTATGCTTCTCCACC-3′
rev 5′-GAGGGGCGCCTGGTGCTCGGGACCC-3′
*Vdr:* fwd 5′-CGCTATGACCTGTGAAGGCTGCAAGGG-3′
rev 5′-GCCAATGTCCACGCAGCGTTTGAGCC-3′
*Xbp1*: fwd 5′-AGCAGCAAGTGGTGGATTTG-3′
rev 5′-CCAAGCGTGTTCTTAACTCCT-3′
*Hmbs* (for normalization): fwd 5′-GACCTGGTTGTTCACTCCCTGAAG-3′
rev 5′-GACAACAGCATCACAAGGGTTTTC-3′

Chromatin immunoprecipitation PCR and qPCR were performed on triplicate samples in all cases, and the data presented as means ± SD. Statistical significance was determined using the unpaired Student’s *t*-test.

### Immunization

Mice stably reconstituted with *Oct2*^+^*^/^*^+^ or *Oct2^−/−^*fetal liver were immunized i.p with 2 × 10^9^ sheep red blood cells (Applied Biological Product Management, Australia) in 100 μl of PBS and sacrificed after 9 days.

## Author Contributions

Lynn Corcoran performed many of the experiments and wrote the manuscript; Dianne Emslie and Tobias Kratina together performed all of the qPCR and protein analyses; Wei Shi performed all bioinformatics analysis for the RNAseq experiments; Susanne Hirsch performed the Slamf1 studies; Nadine Taubenheim contributed to the Xbp1 studies; Stephane Chevrier led most of the flow cytometric analyses.

## Conflict of Interest Statement

The authors declare that the research was conducted in the absence of any commercial or financial relationships that could be construed as a potential conflict of interest.
